# Making Co-Design More Responsible: Case Study on the Development of an AI-Based Decision Support System in Dementia Care

**DOI:** 10.2196/55961

**Published:** 2024-07-31

**Authors:** Dirk R M Lukkien, Sima Ipakchian Askari, Nathalie E Stolwijk, Bob M Hofstede, Henk Herman Nap, Wouter P C Boon, Alexander Peine, Ellen H M Moors, Mirella M N Minkman

**Affiliations:** 1 Vilans Centre of Expertise of Long Term Care Utrecht Netherlands; 2 Copernicus Institute of Sustainable Development Utrecht University Utrecht Netherlands; 3 Human Technology Interaction Eindhoven University of Technology Eindhoven Netherlands; 4 Faculty of Humanities Open University of The Netherlands Heerlen Netherlands; 5 Tilburg Institute for Advanced Studies School for Business and Society Tilburg University Tilburg Netherlands

**Keywords:** responsible innovation, co-design, ethics, decision support systems, gerontechnology, dementia, long-term care

## Abstract

**Background:**

Emerging technologies such as artificial intelligence (AI) require an early-stage assessment of potential societal and ethical implications to increase their acceptability, desirability, and sustainability. This paper explores and compares 2 of these assessment approaches: the responsible innovation (RI) framework originating from technology studies and the co-design approach originating from design studies. While the RI framework has been introduced to guide early-stage technology assessment through anticipation, inclusion, reflexivity, and responsiveness, co-design is a commonly accepted approach in the development of technologies to support the care for older adults with frailty. However, there is limited understanding about how co-design contributes to the anticipation of implications.

**Objective:**

This paper empirically explores how the co-design process of an AI-based decision support system (DSS) for dementia caregivers is complemented by explicit anticipation of implications.

**Methods:**

This case study investigated an international collaborative project that focused on the co-design, development, testing, and commercialization of a DSS that is intended to provide actionable information to formal caregivers of people with dementia. In parallel to the co-design process, an RI exploration took place, which involved examining project members’ viewpoints on both positive and negative implications of using the DSS, along with strategies to address these implications. Results from the co-design process and RI exploration were analyzed and compared. In addition, retrospective interviews were held with project members to reflect on the co-design process and RI exploration.

**Results:**

Our results indicate that, when involved in exploring requirements for the DSS, co-design participants naturally raised various implications and conditions for responsible design and deployment: protecting privacy, preventing cognitive overload, providing transparency, empowering caregivers to be in control, safeguarding accuracy, and training users. However, when comparing the co-design results with insights from the RI exploration, we found limitations to the co-design results, for instance, regarding the specification, interrelatedness, and context dependency of implications and strategies to address implications.

**Conclusions:**

This case study shows that a co-design process that focuses on opportunities for innovation rather than balancing attention for both positive and negative implications may result in knowledge gaps related to social and ethical implications and how they can be addressed. In the pursuit of responsible outcomes, co-design facilitators could broaden their scope and reconsider the specific implementation of the process-oriented RI principles of *anticipation* and *inclusion.*

## Introduction

### Background

In the long-term care for older adults with frailty, caregivers and clients are increasingly being assisted by artificial intelligence (AI)–based technologies [1-5]. AI-based technologies can, for a given set of human-defined objectives, make predictions, recommendations, or decisions influencing real or web-based environments, thereby using machine or human-based data and input [6]. For instance, AI is being used in decision support systems (DSSs) that acquire relevant data about care needs or processes; present the relevant data to users (eg, caregivers); and translate raw data into actionable information, such as alerts, risk assessments, or recommendations about care strategies [7-10]. Notwithstanding the opportunities and advantages, it is broadly acknowledged that the use of AI-based technologies entails societal and ethical implications. The long-term data collection in the context of monitoring older people’s health and well-being and the mediating or even leading role of algorithms in interpreting these data to arrive at care-related decisions pose implications related to, among others, undermining people’s privacy, autonomy, and self-determination; the discrimination and stigmatization of old age; and surveillance capitalism [1,11-15].

Due to the impact technologies such as DSSs have on people’s lives and the potential resistance that might emerge during implementation, an early-stage assessment of their implications is called for. This paper explores and compares 2 of these assessment approaches: the responsible innovation (RI) framework originating from technology studies and the co-design approach originating from design studies. The term RI refers to the aim to ensure the ethical acceptability, societal desirability, and sustainability of innovation processes and outcomes [16,17]. To guide RI into practice, Owen et al [17] suggest that four process-oriented principles should guide technology research and development: (1) anticipation of the potential positive and negative implications; (2) inclusion of users and other stakeholders; (3) reflexivity of actors upon their own practices, assumptions, values, and interests; and (4) responsiveness to insights that emerge during the innovation process.

Co-design can be used as an umbrella term for approaches that actively involve users and other stakeholders of innovations in any stage of the design process to ensure that the outcomes meet their needs [18,19]. It is a commonly accepted approach in the development of technologies to support the long-term care for older adults [20-22]. On a conceptual level, co-design resonates with RI. Both approaches share a focus on developing technologies to match human needs and abilities, similar to research fields such as human factors, human-computer interaction, and cognitive engineering. In fact, co-design has increasingly received attention as a way to support RI [23]. Similar to RI, the co-design approach describes a research and development process in which innovators *inclusively deliberate* and *reflect* on the needs and values of different stakeholders and *iteratively* design and *adapt* innovations based on these insights [23]. However, in contrast to RI, co-design does not explicitly impose on innovators the need to *anticipate* potential societal and ethical implications (henceforth, abbreviated as “implications”). Co-design can yield insights into potential unintended side effects and value creation that stakeholders do not want from innovation, but this is generally not an explicit aim in co-design. Against this background, this paper empirically explores how the explicit *anticipation* of implications can complement co-design.

More specifically, this paper presents a case study on an international collaborative project that focuses on the development of a DSS to support formal caregivers involved in long-term dementia care. A co-design process involving intended users and other stakeholders (henceforth, abbreviated as “users”) is central to the development of the DSS. In addition, a separate line of research of the project under investigation explicitly anticipated implications of using DSSs in dementia care, along with strategies to address these implications, thereby fostering RI in AI-assisted decision-making. This so-called RI exploration largely took place in parallel to (ie, not as part of) the co-design activities and focused on soliciting the perspectives of project members (PMs) rather than those of users. This paper describes the empirical exploration of how the co-design process of an AI-based DSS for dementia caregivers is complemented by the explicit anticipation of implications.

### The Healthy Ageing Eco-System for People With Dementia Project

The case presented in this paper is the Healthy Ageing Eco-system for People With Dementia (HAAL) project, which is part of the European Active and Assisted Living (AAL) program (AAL Europe, 2021; project AAL-2020-7-229-CP). In HAAL, an international consortium comprising care organizations, research institutes, and commercial firms from the Netherlands, Italy, Taiwan, and Denmark collaborates on the co-design, development, testing, and commercialization of a DSS that is intended to provide actionable information to formal caregivers of people with dementia, with the aim of reducing their workload and increasing the quality of care [24]. The DSS developed in HAAL concerns a dashboard that integrates various types of data about the physical activity, eating and sleeping patterns, cognitive functioning, mood, social contact, and medication intake of people with dementia. These data can be collected via several digital technologies (henceforth, “HAAL technologies”) throughout various stages of dementia. Besides integrating the data from HAAL technologies into 1 dashboard, possibilities to provide caregivers only the most relevant data in the form of summary overviews, alerts, predictions about emergency situations, and recommendations about care strategies were explored. To this end, both preprogrammed, rule-based algorithms and data-driven algorithms rooted in machine learning are used to process data.

With these predefined directions as a starting point, a series of iterative co-design activities involving dementia caregivers, or more correctly “proxy users” who represent these eventual users (see the study by Stewart and Hyysalo [25]), and other stakeholders were organized to feed the actual design and development of the dashboard. The co-design activities focused on exploring the relevance and possibilities of translating the data from HAAL technologies into useful information and prioritizing data that are relevant to be presented in the dashboard [24,26]. In addition, the co-design activities focused on determining functionalities of the dashboard and designing and evaluating different pages of the dashboard’s user interface.

The RI exploration in HAAL, which took place largely in parallel to the co-design activities, initially focused on raising PMs’ general awareness about RI and exploring their perspectives on both positive and negative implications of using the HAAL dashboard, along with strategies to address these implications.

## Methods

### Overview

**Figure 1:**
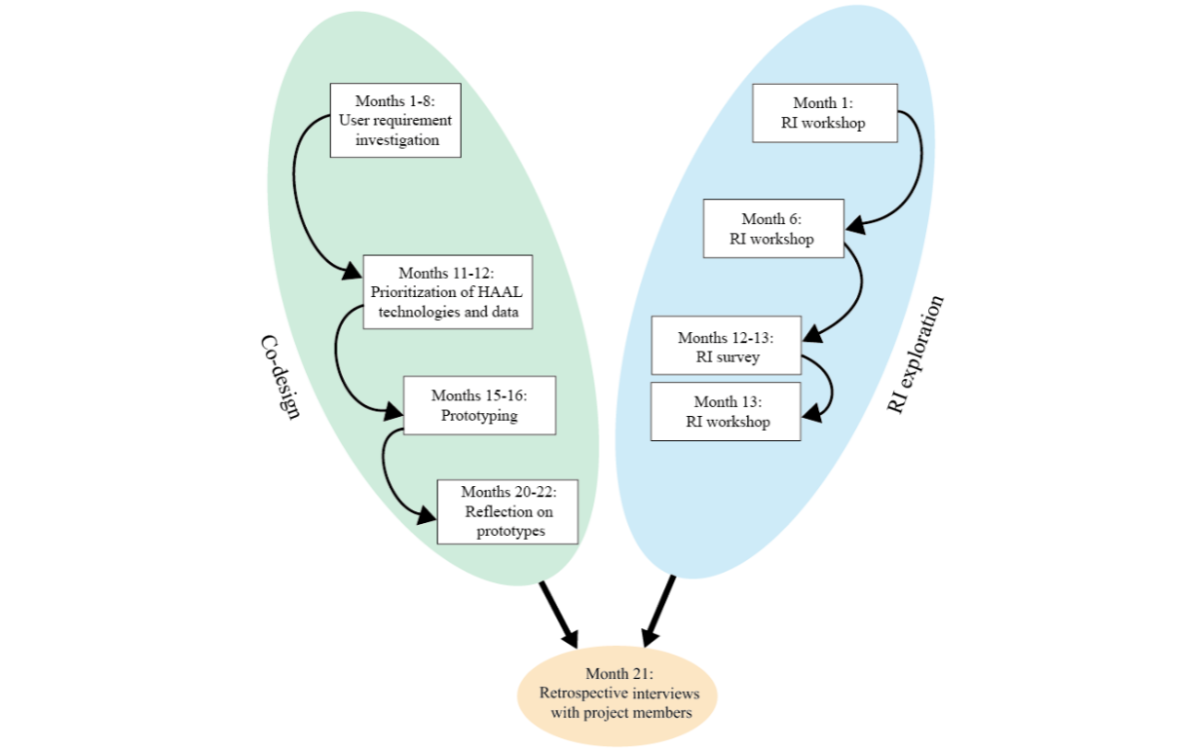
Timeline of the co-design process, responsible innovation (RI) exploration, and retrospective interviews. "Month" refers to the month (count in project) in which the activity took place. HAAL: Healthy Ageing Eco-system for People With Dementia.

For this case study, results from the co-design process and RI exploration within the HAAL project were incorporated and analyzed. In addition, retrospective interviews were held with individual PMs to reflect on the co-design process and RI exploration. Because the co-design process and RI exploration were largely organized in parallel, the HAAL project provided sufficient data within a specific time and context to perform a retrospective analysis on how the explicit anticipation of implications can complement co-design. Figure 1 shows a timeline of activities.

### Co-Design Process

Table 1 describes the 4 specific steps taken in the co-design process. The co-design activities in HAAL were conducted in 4 countries: the Netherlands, Italy, Taiwan and Denmark. The organizations from Denmark are unsubsidized partners in the HAAL project and did not participate in co-design steps 3 to 4. Despite differences in dementia care systems across these countries, such as types of caregivers involved in home-based and institutionalized care settings, formal caregivers of people with dementia were perceived as the primary target group for (using) the dashboard in all countries. Hence, a variety of formal caregivers of people with dementia, such as (homecare) nurses, case managers, psychologists, psychotherapists, social workers, and specialists in the care of older adults, were involved in the co-design activities. In addition, other stakeholders, such as innovation staff, data analysts at care organizations, and people working in (care) alarm centrals, were involved in some steps of the co-design process to broadly explore requirements for the dashboard. As indicated in Table 1, two intermediate steps were taken without the direct involvement of users. Further, at the end of step 4, participants were implicitly asked about RI-related themes (autonomy and transparency). Throughout the co-design activities, data were collected in the form of notes, audio and video recordings, photos, drawings, and (web-based) canvasses and by conducting surveys.

**Table 1:** Steps taken in the co-design process.

Step	Methods	Research focus	Participants
1^a^	Focus group sessions (3 web based, 1 hybrid, and 16 physical)	User requirement investigation: insights were gathered into different stakeholders’ attitudes toward the HAAL^b^ technologies, their ideas about the added value and functionalities that are envisioned in the integration of these technologies in 1 dashboard, and for which stakeholders such a dashboard may be most relevant.	Nurses, day-care workers, psychologists, physiotherapists, technical stakeholders, innovation managers and directors of care organizations, representatives from various municipalities, people with dementia, and informal caregivers (n=146; the Netherlands: n=18, 12.3%; Italy: n=18, 12.3%; Taiwan: n=108, 74%; Denmark: n=2, 1.4%).
2	Demonstration, try-outs, and survey (7 physical and 1 hybrid)	Prioritization of HAAL technologies and data: based on the MoSCoW^c^ technique, all HAAL technologies and corresponding data were categorized into 4 ascending categories (must have, should have, could have, and won’t have this time), indicating what best fits the needs of people with dementia and their caregivers [27,28]. After a demonstration and try-outs of the HAAL technologies, participants completed a digital prioritization survey.	Nurses, day-care workers, psychologists, physiotherapists, data specialists, and innovation staff and directors from care organizations (n=48; the Netherlands: n=6, 12%; Italy: n=9, 19%; Taiwan: n=30, 62%; Denmark: n=3, 6%).
3^d^	Co-design sessions (3 physical and 2 web based)	Prototyping: three dashboard pages were preselected to be co-designed with participants: (1) client profile with detailed information on specific clients, (2) overall list of clients, and (3) an overview of urgent situations. This resulted in insights (ie, through sketches and design by participants) into the kind of information to be displayed in the dashboard and how the information could be visualized. Finally, the results were compared with those of the preliminary mock-up.	Data specialists and innovation staff, including part-time nurses (n=21; the Netherlands: n=6, 29%; Italy: n=4, 19%; Taiwan: n=11, 52%).
4	Usability study (8 physical sessions, including survey)	Reflection on prototypes: insights were gained into the usability and heuristics of the clickable mock-up. More specifically, after first performing 6 tasks in the mock-up, participants completed a survey. In the survey, the HUBBI^e^ questionnaire [29] was used to determine usability, and heuristics were evaluated using the issue categories of Bastien and Scapin [30] and Nielsen’s severity ranking [31]. After completing the survey, participants engaged in a group discussion on the overall added value and functioning of the dashboard. At the end of the discussion, participants were also asked to reflect on 2 RI^f^ themes: Autonomy: do you think the degree to which the dashboard guides your decision-making as a caregiver is adequate, too low, or too high, and why do you think so? Transparency: what would you choose, if you had to choose between either the accuracy of the information (ie, recommendations) provided by the dashboard or the understandability of the information, and why would you choose it?	Formal caregivers, digital care ambassadors, alarm centralists, and innovation staff (n=33; the Netherlands: n=9, 27%; Italy: n=14, 42%; Taiwan: n=10, 30%).

^a^Intermediate step: after analyzing results from step 1, user personas and desired dashboard functionalities were defined and translated into a preliminary mock-up for the dashboard (iteration 1). The motivational goal model of Taveter et al [32] was used for this translation.

^b^HAAL: Healthy Ageing Eco-system for People With Dementia.

^c^MoSCoW: must have, should have, could have, and won’t have this time.

^d^Intermediate step: after analyzing results from step 3, insights about user requirements were again plotted on the motivational goal model to define design requirements. These design requirements were used to translate the preliminary mock-up into a clickable mock-up (iteration 2).

^e^HUBBI: eHealth usability benchmarking instrument.

^f^RI: responsible innovation.

### RI Exploration

The RI exploration was primarily based on a qualitative survey among PMs, which was preceded by 2 workshops and followed by a third workshop with PMs. The first 2 workshops with PMs were held in a hybrid setting (web based and physical) during collective consortium meetings. The goal of the first workshop was to explain the notion of RI to PMs and discuss their thoughts about the relevance of and ways to address RI in HAAL. In the second workshop, based on the guidance ethics approach of Verbeek and Tijink [33], potential positive and negative implications of using the envisioned HAAL dashboard were explored, along with ways to address these implications.

Next, a dedicated qualitative RI survey was developed and conducted among PMs (Multimedia Appendix 1). The goal of the RI survey was to reveal PMs’ viewpoints on how to responsibly develop AI-based analytical functionalities and the dashboard user interface in the HAAL project. The survey first explained that AI, as in the HAAL dashboard, provides opportunities for descriptive, diagnostic, predictive, and prescriptive analyses with differing levels of complexity and automation [34,35]. Next, questions were asked in relation to 2 distinct imaginary scenarios that outline different roles for AI within the HAAL dashboard. The first scenario (A) described a descriptive and largely rule-based dashboard through which users can assess the data from HAAL technologies and how the situations of clients have changed over time. This scenario was inspired by the dashboard that was aimed to be developed in the HAAL project. The second scenario (B) took a more speculative turn and described a proactive and partially self-learning dashboard that automatically translates the data into diagnostic, predictive, and prescriptive information to prompt caregivers to take certain actions. The scenarios were used as input to inspire respondents about directions the project could take in terms of developing AI and to enable them to articulate their expectations and considerations regarding the opportunities and implications of an advanced AI-based DSS (see also the study by Noortman et al [36]). After presenting each scenario, questions were asked about the positive and negative implications of using the respective dashboard. Thereafter, respondents were asked which scenario they preferred in terms of ethical acceptability, societal desirability, and technical feasibility and why they preferred it. Next, the survey introduced six principles for responsible AI innovation, adopted from guidelines from the World Health Organization: (1) protecting human autonomy; (2) promoting human well-being and safety and the public interest; (3) ensuring transparency, explainability, and intelligibility; (4) fostering responsibility and accountability; (5) ensuring inclusiveness and equity; and (6) promoting AI that is responsive and sustainable [37]. Respondents were asked how these principles might be relevant to and could be applied in the HAAL project. The survey was completed by 12 respondents representing 7 different organizations from all 4 countries. In addition, 5 respondents partially filled in the survey anonymously.

Finally, the RI survey was followed by a third hybrid workshop in which PMs were invited to jointly discuss what they learned from answering the RI survey.

### Retrospective Interviews With PMs

In addition to the co-design activities and RI exploration, semistructured interviews were held with 6 PMs: 4 co-design facilitators (n=1, 25% working in the Netherlands; n=2, 50% working in Taiwan; and n=1, 25% working in Italy) and 2 software developers (working in Italy). The goal of the interviews was to uncover possible rationales behind the co-design process, choices made throughout the co-design process, and input given by co-design participants. All interviews lasted between 30 and 40 minutes and were fully transcribed by a professional transcription service.

### Analysis

The analysis of data was performed by DRML, SIA, NES, and BMH. The data collected during the co-design activities and RI exploration were first analyzed independently by these 4 researchers. While the co-design data were previously analyzed by HAAL PMs to learn about the dashboard requirements, they were analyzed again for the purposes of this paper. Taking the 6 responsible AI principles from the World Health Organization guidelines [37] as a starting point, the researchers performed an inductive thematic analysis [38] to uncover conditions for the responsible design and deployment of the HAAL dashboard, including potential negative implications and strategies to address them. In doing so, they examined how certain insights regarding these conditions emerged in the co-design activities, the RI exploration, or both. In other words, the analysis focused, first, on identifying themes common within and between the co-design and RI exploration results and, second, on examining how the results from the RI exploration complement those from the co-design activities, or vice versa, in terms of RI. Subsequently, the transcripts of the retrospective interviews were analyzed independently by DRML, SIA, and BMH to uncover new conditions for RI and explore the complementarity between the co-design process and RI exploration. An additional focus was on why certain insights about conditions for RI may have emerged less explicitly in either the co-design process or the RI exploration. While analyzing the data, the researchers applied open coding and kept track of their reflections by writing them down as memos. After the data were independently analyzed by the researchers, the findings and memos were regularly discussed and reviewed by the researchers to reconcile major discrepancies in the coding and to reach agreement on the final coding scheme. Both physical and digital meetings were held to ensure the consistency of the analysis and reach convergence.

### Ethical Considerations

The authors of this study followed the guidelines in the Declaration of Helsinki and the Dutch code of conduct for scientific integrity. Ethical approval for the interviews, not subject to the medical scientific research act involving human subjects, was granted by an independent board of the lead author's department (Vilans), including a privacy officer and legal expert [39].

For each co-design step, general information about the goal and procedure was provided, and the participants were asked to read and sign an informed consent form. The original consent covers secondary analysis of the data for the purposes of this study. The data gathered through the co-design steps and RI exploration were pseudonymized before analysis. Study participants did not receive any financial compensation.

## Results

### Overview

Seven overarching and interlinked themes representing conditions for the responsible development and deployment of the HAAL dashboard were extracted: (1) develop a proactive dashboard, (2) prevent cognitive overload, (3) protect privacy, (4) provide transparency, (5) empower caregivers to be in control, (6) safeguard accuracy, and (7) train users. We explicate how insights related to each theme emerged in the co-design activities, the RI exploration, or both. In addition, insights from the interviews with PMs are provided. In doing so, for each theme, we discuss how the explicit anticipation of implications (ie, the RI exploration) complements the co-design process in the HAAL project. Textbox 1 excerpts the results.

**Analysis of complementarities between the co-design process and responsible innovation (RI) exploration per theme.**

1. Develop a proactive dashboard
  The co-design results clearly indicate a perceived need for a proactive dashboard and provide concrete arguments to this end. The RI exploration also indicated the need for a proactive dashboard, albeit with less concrete arguments. Besides, limitations were raised regarding the short-term feasibility of a proactive dashboard.
2. Prevent cognitive overload
  The co-design process and RI exploration yielded similar insights, that is, that too much data in one place would overload caregivers’ cognitive workload and that focus of the dashboard should be on providing actionable and only the most relevant information. However, this insight only emerged late in the co-design process (step 4 of 4).
3. Protect privacy
  The need for privacy protection emerged strongly in the co-design process, and participants clearly pointed to the need for a proactive dashboard in privacy terms. The theme was discussed only briefly in the RI exploration, although some practical suggestions were provided, such as the use of encryption and passwords.
4. Provide transparency
  While the importance of the transparency of the dashboard’s information emerged in the co-design process, practical suggestions on how to provide transparency (eg, training users in correctly interpreting information and explanations) were given only in the RI exploration.
5. Empower caregivers to be in control
  The main contribution from co-design was the proposition to gradually expand the application of artificial intelligence (AI) functions in practice so that users can get used to an increasing role of AI. In comparison, the RI exploration yielded more in-depth insights and suggestions. The RI exploration stressed that it is important for caregivers not to become too reliant on the results of AI and to have a critical mindset and keep the context in mind.
6. Safeguard accuracy
  During co-design, the importance of accurate dashboard information was mentioned but not discussed in depth. In the RI exploration, concrete suggestions were made to ensure accuracy, such as including feedback buttons for users.
7. Train users
  The importance of training, also in relation to other themes such as empowering caregivers to be in control and safeguarding accuracy, frequently appeared in the RI exploration but was raised by only one of the participants in the co-design process. In the RI exploration, suggestions were also provided regarding the focus of training, for instance, on creating awareness about the mediating role of AI in decision-making.

### Theme 1: Develop a Proactive Dashboard

The co-design participants generally agreed that the HAAL dashboard should support decision-making proactively, by actively generating and pointing users to relevant insights, rather than passively, by merely showing data from the HAAL technologies. In contrast, the results from the RI survey showed varying viewpoints among PMs regarding the dashboard’s required level of proactiveness with regard to supporting decision-making.

Co-design steps 1 and 2 showed that the data from HAAL technologies could be potentially useful for both daily caregivers and caregivers who are less frequently involved (eg, general practitioners). In these co-design steps, there was limited reflection on the possibilities of a dashboard beyond data integration. However, in co-design steps 3 and 4, most participants expressed an interest in a dashboard that also interprets data to provide new information and inspire users. That is, participants suggested that the dashboard should provide insights into or predictions about outliers from usual patterns and distinguish between urgent (eg, a fall) and nonurgent (eg, a deviation in sleeping pattern) outliers to prompt caregivers to take appropriate action. As one of the caregivers at a Taiwanese care center argued, “What I would like is an alert service, more centered on urgency than on daily, routine patient follow-up*.”* In addition, the dashboard was seen as a way to encourage caregivers to consider signs that might otherwise have been neglected or perceived too late. Besides, some participants of co-design steps 3 and 4 proposed that the dashboard could provide recommendations on how to prevent or address certain deviations from usual patterns.

In the RI survey, most PMs shared pros and cons related to both a descriptive dashboard (scenario A) and a proactive dashboard (scenario B). Most PMs argued that a proactive dashboard could potentially add the most value, especially in terms of enhancing prevention and reducing caregivers’ cognitive load (see also theme 2). At the same time, all PMs expressed doubts about the feasibility of developing a proactive dashboard due to the complexity and relatively limited time span of the HAAL project. Some PMs stressed that the initial acceptance and adoption of a proactive dashboard by caregivers might be low, arguing that the more proactive the dashboard is, the more it may infringe on job satisfaction. As one of the PMs explained, “Caregivers might enjoy the part in their work where they investigate the status of the client, and this is then (partially) taken over by machines.” However, although market introduction was questioned, some PMs advocated exploring possibilities for and experimenting with the more progressive concept of a proactive dashboard to iteratively learn and generate ideas and lessons for future research and development. In an interview, one of the PMs explained, “We know that we could do bigger, smarter things with AI, but you cannot start with high-level AI...But I think that these kinds of projects are useful also to build knowledge and literacy, by making people consider what technology and artificial intelligence could do*.*”

### Theme 2: Prevent Cognitive Overload

The need to prevent cognitive overload was another recurring argument for developing a proactive dashboard in both the co-design process and RI survey. In co-design step 4, it was stressed by multiple participants that too much data or information in one place could exceed caregivers’ cognitive load and cause problems regarding the prioritization of which client, or what aspect of a client’s life, needs attention first. Similarly, in the RI survey, PMs suggested multiple times that a descriptive dashboard may require additional time for caregivers in terms of checking the data, rather than save time, and increase mental strain. As one of the PMs stated, “Adding more data in one place without elaborating on it would not really reduce the caregiver burden.”

### Theme 3: Protect Privacy

While privacy was a prominent theme throughout all co-design steps, it was only briefly discussed in the RI exploration. During co-design step 3, multiple participants suggested that from a privacy perspective, a (proactive) dashboard that provides only the most relevant data patterns, notifications, and alerts may be preferred over a (descriptive) dashboard that directly discloses all data about the evolving status of clients in relation to various indicators. This link between the need for a proactive dashboard (scenario B) and privacy concerns was not discussed in the RI survey.

Further, privacy concerns raised in the co-design activities were related to the storage of large amounts of data collected about people with dementia and how these data would be handled. As one of the participants stated, “A lot of personal information is gathered, so you can get to know a lot about people*.*” In line with this, most participants stated that compliance with the European General Data Protection Regulation should be ensured, and some practical suggestions were made, for instance, to show the client’s home address or room number in the dashboard rather than their names in case of alarms.

The importance of privacy protection was mentioned by various PMs in the first 2 RI workshops, but in the RI survey, only 3 (18%) of the 17 PMs provided input on privacy issues. One of the PMs stated that ways must be found to balance the benefits of large-scale and long-term data collection (eg, in terms of prevention) with downsides such as a feeling of intrusion. Complementary to the co-design process, PMs also provided practical suggestions on privacy protection in the RI survey, such as using a private log-in to the dashboard for caregivers, encryption, or even facial recognition to protect data.

Another privacy concern, raised during co-design step 3, was data accessibility. Several participants reported about who should have access to the dashboard. Some participants proposed that access should be limited to specific caregivers with the specific assignment to learn from the dashboard. In contrast, others reported that all caregivers, including informal carers (eg, family), should have access to the dashboard, if desired. There was no consensus among participants about whether a distinction should be made between different users who are able to see different client data.

### Theme 4: Provide Transparency

In co-design step 4, participants proposed that a condition for the use of a proactive dashboard is that users need to understand the reasons (eg, data patterns) behind information provided by the dashboard. In this respect, one of the PMs discussed in an interview that caregivers should not be overloaded with too many details about how specific dashboard information comes about (see also theme 2). In contrast, some co-design participants stressed that users should always be able to examine all data from the different HAAL technologies. Hence, this could be in conflict with the previously discussed insight from co-design that making all data available may be less preferable from a privacy perspective (see also theme 3).

The co-design participants also made various remarks regarding the context specificity of transparency needs. Multiple participants expressed that a need for transparency may not always, or for every user, mean the same. For instance, in case of alarms about certain urgent situations, it may be irrelevant or even distracting to immediately show all data that triggered the alarm. However, users may want to view all the data at a later stage to gain insights into the context and possible causes for the urgent situation, for instance, for training and prevention purposes. A similar insight was raised in the RI exploration, where it was, for example, suggested that in-depth explanations could be provided but only after users ask for it, for instance, by clicking through.

Further, during co-design step 4, it was suggested that once caregivers have built a certain level of trust in the dashboard, less detailed explanations clarifying how the dashboard reaches its conclusions might be sufficient. However, as one of the PMs added in an interview, in the long run, excessive trust might lead to caregivers making certain decisions too easily based on the dashboard’s information without critical reflection: “The long-term risk is that users end up trusting the system too much*”* (see also theme 5).

Although co-design participants highlighted the importance of transparency in HAAL, they did not provide practical suggestions about ways to provide transparency. In the retrospective interviews, various possible explanations were given. For instance, 2 PMs argued that issues such as transparency may have been discussed with limited depth throughout co-design because they pertain more to the backend of the system (ie, algorithms and web services) than the front end (ie, interface) with which users directly interact and because participants may place a certain degree of trust in developers to deal with such issues. Besides, 2 PMs discussed that it may have been hard for co-design participants to formulate requirements regarding transparency during early phases of design because the dashboard concept was still relatively abstract. As suggested, gaining in-depth insights into issues such as these may be easier when practically demonstrating and testing the dashboard in field tests, as users can then actually experience the system and its limitations.

While practical suggestions on providing transparency in HAAL were absent in the co-design results, they were discussed in the RI survey. For instance, PMs suggested (1) showing which specific data were included by algorithms to provide certain information; (2) creating abstractions easy to understand for users to explain the logic behind data analyses, for instance, by giving explanatory examples of common use cases; and (3) training users in interpreting the information and their explanations (see also theme 7).

### Theme 5: Empower Caregivers to Be in Control

It was raised in co-design step 4 that people should be in charge of decision-making, regardless of whether human decisions are in line with the dashboard’s information. In the same line, multiple PMs argued in the RI survey that people (ie, caregivers) should always be making the final decisions, and they should make these decisions only after carefully valuing the dashboard’s information in light of the specific context. It was also suggested during co-design that caregivers may at first instance not be ready yet to get extensive advice from a dashboard. A gradual expansion of AI-functions in real practice was suggested. For instance, in the beginning, the dashboard could provide only generic insights (eg, patterns), alarms, and predictions. In a later stage, when reliability has improved and trust in and experience with the system have been gained, recommendations or conclusions about follow-up steps could be provided. Apart from the above, the importance of people making the final decisions was not further reported by co-design participants.

In contrast, the importance of caregivers being and remaining to be in control of decision-making was more prominent in the RI exploration. In the RI survey, 3 PMs suggested that the long-term use of a proactive dashboard might slowly deprive the intuition of caregivers and maintain an automated and predefined focus whereby one might overlook the person (ie, person with dementia) behind the data. One of the PMs even stated, “There may be a tendency to rely more on AI than own observations and assessments because ‘the computer is always right.’” To encourage caregivers to make autonomous decisions while using the dashboard, training was put forward as an important factor by several PMs (see also theme 7).

### Theme 6: Safeguard Accuracy

The importance of accurate dashboard information was reflected to a limited extent in the co-design process. During all co-design steps, participants reported a couple of times that the accuracy of the data and data analyses should be regularly evaluated. However, in an interview, a PM suggested that co-design participants mainly shared this requirement as a general condition that must be met before the dashboard could be put into practice, rather than giving concrete ideas on how to achieve this.

The importance of accurate dashboard information and ways to achieve this were more prominently discussed in the RI survey. Multiple PMs argued that information provided by the dashboard should not lead to any faulty judgments by caregivers and that both the data and the algorithms processing data should, therefore, be accurate, without significant biases. For instance, one of the PMs stated, “The dashboard should not give unnecessary warnings to caregivers because the false warning could stimulate the caregivers to impose unnecessary boundaries to people with dementia*.*” One of the PMs explicitly linked accuracy to being sensitive toward the diversity among clients and suggested that the dashboard be fed with data from heterogeneous clients to reduce bias. In contrast to the co-design process, PMs also provided practical suggestions about particular ways of involving users to safeguard accuracy, such as enabling users to (1) provide feedback on data or insights through a button, (2) personalize certain thresholds for alarms to the individual client, (3) keep track of their responses and follow-up actions on the dashboard’s information, (4) report nonplausible suggestions and malfunctions, and (5) periodically evaluate the dashboard’s functioning. Again, training was put forward as an important factor in this case for users to be able to be involved (see also theme 7).

### Theme 7: Train Users

During the co-design activities, one of the participants commented that the proper use of the dashboard would require training and practical learning. In the RI survey, multiple PMs pointed out that training users is an important measure to tackle challenges related to the autonomy of users and the accuracy of the dashboard’s information (see also themes 5 and 6). It was suggested that the training should focus on making the users become acquainted with the HAAL technologies; data types; and information provided by the dashboard, including underlying data analyses, and on understanding the impact that the use of the dashboard might have on decision-making. One of the PMs said, “Caregivers should be taught that they will always in some degree be influenced by the information on the dashboard, and be recommended to make their own judgements first.” Another PM argued that training should prevent caregivers to become overreliant on the dashboard. In addition, training was suggested to prepare some users for active involvement in maintaining the accuracy of the dashboard information (see also theme 6).

## Discussion

### Principal Findings

This paper empirically explores how the co-design process of an AI-based DSS for dementia caregivers is complemented by the explicit anticipation of implications. A total of 7 overarching and interlinked themes representing conditions for the responsible development and deployment of the DSS were extracted: develop a proactive dashboard, prevent cognitive overload, protect privacy, provide transparency, empower caregivers to be in control, safeguard accuracy (eg, by reducing false positives), and train users. Because these conditions are interlinked, it is essential for various actors, including developers and users of the DSS, to work together to cohesively address them in practice. Moreover, some conditions, such as to develop a proactive dashboard and empower caregivers to be in charge or to provide transparency through detailed information and prevent cognitive overload, can be at odds with each other and need to be carefully balanced. To gain a deeper understanding about appropriate and responsible levels of proactivity by the DSS, where the contributions of AI and human input in decision-making are balanced, future studies could expand upon prior research in fields such as human factors by exploring and contextualizing notions such as automation bias [40,41] and human automation coordination [42,43] in the context of AI-assisted decision-making in long-term dementia care. Scenarios that may lead to excessive reliance on the automated execution of functions, such as AI-driven data interpretation, could be anticipated, and strategies could be devised to mitigate such scenarios [40].

As our analysis points out, the general expectation of both co-design participants and PMs was that a dashboard that proactively supports decision-making would be most valuable to dementia caregivers. To this regard, the perspectives of co-design participants were fairly aligned; there was a consensus that the dashboard should not show all available data from care technologies. Rather, it should focus on information about significant changes in the data that, for instance, indicate a deterioration of well-being. AI itself was positioned as a technical fix (see also the study by Wehrens et al [44]) to mitigate specific risks related to the remote technology-based monitoring of people with dementia, that is, the infringement of clients’ privacy and cognitive overload of caregivers. This is in line with previous studies that show that too much information [45-47] and insufficient time can lead to information overload [48]. The same suggestion of using AI to actually support the responsible embedding of technology in care practice was also found in a scoping review on practical approaches to responsible AI innovation in the context of long-term care [49]. In comparison to the co-design results, the perspectives of PMs in the RI exploration were less unanimous; some PMs shared doubts about the short-term feasibility and acceptance of a proactive dashboard. This discrepancy between results may have been owing to the co-design process being focused on exploring opportunities for innovation, while the RI exploration explicitly invited PMs to reflect on opportunities as well as risks of AI-based analytical functionalities.

Throughout both the co-design process and the RI exploration, various conditions were defined for the responsible development and deployment of a proactive DSS. Similar conditions emerged in the co-design process and RI exploration. However, despite considering and addressing usability requirements, such as minimizing memory load [31,50], in the co-design process, co-design participants generally went into less detail. Compared to PMs in the RI exploration, co-design participants provided fewer practical suggestions on how to meet the RI conditions, except for conditions related to privacy protection. In addition, multiple conditions (ie, preventing cognitive overload, empowering caregivers to be in control, and safeguarding accuracy) emerged in a relatively late stage of the co-design process, once prototyping and reflection on prototypes stood central. Relevant input on implications and conditions for RI emerged more naturally in these phases of co-design, regardless of 2 RI questions related to autonomy and transparency being asked at the end of the last co-design step. Again, these differences in results could potentially be explained by the focus of co-design activities being mainly on opportunities, while the RI exploration was focused on both opportunities and risks.

Hence, the explicit anticipation of implications (ie, the RI exploration) was found to complement the insights from the co-design process in the project under investigation. At the same time, a number of deficiencies can be mentioned regarding the insights that have been gained about social and ethical implications of the DSS. For instance, potential tensions were found between conditions set by different co-design participants. More specifically, to protect privacy, some co-design participants proposed to limit access to information provided by the DSS to specific caregivers. Other participants advocated more transparency and data availability. It is premature to draw conclusions from such contrasting insights. However, it can be stated that insufficient insights were gained into people’s individual views on such matters, the interrelatedness of conditions, and potential trade-offs between them. Further, it stood out that both the co-design process and RI exploration yielded limited insights into the dependency of different conditions on context (eg, time, place, and culture). Although it was indicated that trust in the dashboard and transparency needs may change over time, limited insights were gained into how conditions for RI may depend on other contextual factors, such as place and culture. Despite the co-design activities being carried out in multiple countries, no cross-country differences in conditions for the responsible design and deployment of the dashboard were found.

### Practical Implications

As argued by Fischer et al [22], differences regarding who is involved in the co-design of care technologies, and how, when, and why they are involved, result in different types of outcomes. To this respect, we discuss 4 considerations that designers and co-design facilitators could take into account to increase the potential for co-design processes to contribute to ethically acceptable, societally desirable, and sustainable deployments of AI-based care technologies.

First, one could strive for balanced attention on both positive and negative implications throughout co-design processes. The co-design process in this case study was focused mostly on functional (ie, what the technology must do) and nonfunctional (eg, usability and reliability) requirements. However, rather than merely eliciting information on the needs, preferences, and requirements of users, co-design processes should go back and forth between needs and opportunities for innovation on the one hand and associated implications on the other hand. In addition, RI necessitates striking a balance in co-design practices between focusing on design aspects, such as usability and esthetics, and considering ethical and social implications. Adhering to specific design standards holds importance to meaningful field tests and the implementation of innovations in practice. However, excessive emphasis on these aspects during early phases of innovation may detract from fostering the innovation’s desirability and acceptability. Although research and development projects that *integrate* anticipatory elements into co-design may yield more in-depth insights and be able to more flexibly adapt to insights than projects that anticipate implications separate from the co-design process, a few remarks can be made here. For instance, implications of innovation may need to be anticipated and addressed not only as part of co-design but also in parallel to and beyond the co-design process through methods such as impact assessments, ethical reviews, and foresight exercises. Besides, caution should be exercised to prevent co-design processes from becoming dominated by the anticipation of long-term and wider societal implications, as this may go at the expense of fast iterative design cycles exploring and addressing requirements and direct benefits for users. Further, Sumner et al [21] argued that co-design may require the commitment of a significant amount of time and resources and that some projects may have to rationalize limited resources. Naturally, the same applies to anticipating implications as part of or in parallel to co-design.

Second, one could engage with the perspectives of people who are willing and able to imagine how their interests and their role as users of technology evolve over time (ie, future users), rather than merely involve people from contemporary care practices in co-design. Innovators should not just examine the needs of current users because they may then be insufficiently able to respond to future needs [51]. For instance, in the context of the HAAL project, which was investigated in this study, this could concern the involvement of progressive and technology-savvy dementia caregivers who reflect on how the adoption of increasingly advanced DSSs and other AI technologies will change their work.

Third, one could deliberate on which stakeholders, apart from users, should actually participate in co-design and regularly evaluate how their views guide the underlying direction of innovation. Due to the focus of co-design often being on the needs, expectations, and contexts of individual users, innovators may fail to address potential negative implications, especially implications for other stakeholders or in the long run [52]. Accordingly, it might be relevant to involve certain stakeholders such as intermediary user organizations or social advocacy groups in co-design to articulate societal demands and consider societal implications from a systemic perspective [25,53,54]. For instance, in the context of the HAAL project, this could concern involving nongovernmental organizations that are committed to the privacy interests of older people.

Fourth, one could not only invite but also actively enable users to contribute to the anticipation of implications in co-design. As users are often no experts in (responsible) innovation, they may have difficulties in explicating implications and how they could be addressed, even if explicitly asked for. In this case study, it became more natural for co-design participants to come up with implications in the later phases of co-design (ie, steps 3 and 4) when the dashboard concept had become more tangible. To enable the anticipation of implications *early* in the co-design process, it may be useful to develop inspirational tools that use, for instance, examples of negative impacts of AI technologies [55], envisioning cards [56], or design fiction [36,57] to evoke consideration of the possible intended and unintended short- and long-term effects of future technologies. In addition, in the context of AI-based innovation, one could ensure through training that co-design participants have a basic understanding of what AI can do and how its behavior may be unpredictable and change over time while accumulating data [58,59].

In sum, for co-design processes to result in more RI outcomes, designers and co-design facilitators may need to broaden their scope and reconsider the specific implementation of the process-oriented RI principles of *anticipation* and *inclusion* [17,60]. Even though there are still many uncertainties about the potential uses and consequences of technology during early phases of co-design and before users can “experience” the technology in practice, the anticipation of implications with users ideally starts early, before the technology design has been locked in and change becomes difficult, time-consuming, and expensive [61]. Besides, anticipation should be a recurring element of the innovation process, as people’s values and perspectives on what is *responsible* may evolve over time and under the influence of technological innovation [62].

### Limitations and Suggestions for Future Research

Given that this paper studies merely a single case, our aim is not to generalize, but rather to illustrate a typical co-design process of an AI-based technology to support the care for older adults and contribute to building a nuanced view on the relation between co-design and RI [63]. Although we use a broad definition for co-design, we acknowledge that there are multiple ways, methods, and instruments to integrate users into the innovation process [21]. Therefore, our findings about the role of anticipating implications in co-design are not generally applicable to co-design. For instance, it is plausible that projects that adopt the value-sensitive design approach yield different results, as this approach aims to explicitly consider the values of users and other stakeholders and how these values are affected by the envisioned technology [64-66]. In other words, some approaches to co-design may in themselves impose on facilitators to explore the values at stake and thereby the implications of innovation. Future research could examine to what extent such approaches support RI.

Further, we recognize that there are limitations to the RI exploration that was part of our study and thus to the insights gained into conditions for the responsible development and deployment of DSSs in dementia care. Our RI exploration initially focused on the perspectives of PMs to stimulate and facilitate whole-team participation in exploring how RI could be addressed throughout the HAAL project. The underlying assumption was that RI cannot be prescribed to innovators but needs to be conceptualized and addressed “in context” by those who actually perform the research, design, development, and testing with users [67,68]. However, soliciting PMs’ perspectives provided neither a complete nor necessarily an accurate picture about implications and ways they can be addressed. To this end, future studies could consider embedding trained ethicists in the research team who can provide *top-down* guidance and inspiration (eg, contextualized ethics principles) during *bottom-up* engagement with users and other stakeholders [49,69]. Besides, future research could explore the perspectives of users on RI in the context of AI-based care technologies, such as DSSs, for instance, what values come to matter most to them, what positive and negative implications they foresee, how they perceive the urgency of (other) known implications in their context, and how they look at certain strategies to address implications (eg, see the study of Lukkien et al [70]). In doing so, the perspectives of stakeholders from different care contexts (eg, care organizations or countries) can be captured with sufficient detail and be compared to learn how to account for the context specificity of values in technology design and deployment [71,72]. In addition, the perspectives of people with dementia should be clarified, even when they are only a passive user of the technology (as is often the case with DSSs), and despite these people often having difficulties in expressing their needs [73,74].

Finally, even though all co-design activities and the RI exploration had already been completed by the time the objectives for this case study were established, the RI exploration had a minor effect on the co-design process. For instance, some co-design researchers were also participants in the RI exploration, which could have affected the co-design activities. Besides, at the request of DRML (who led the RI exploration), the usability study (co-design step 4) included 2 RI-related questions. In our results, we explicated that co-design participants already discussed more implications before these 2 questions were asked. Without this minor effect, there may have been a greater knowledge gap between the results from the co-design process and RI exploration in HAAL. However, to gain more robust results into the role of the anticipation of implications in co-design, future research could study co-design processes completely separately from an exploration of associated implications.

### Conclusions

In this paper, we explored how the co-design process of an AI-based DSS for dementia caregivers is complemented by the explicit anticipation of social and ethical implications. Co-design is an essential means to feed the development and deployment of AI-based care technologies with insights about needs of targeted users and collectively translate these needs into requirements for technology design. Besides, as found in this empirical study, certain implications and strategies to address these implications may be naturally anticipated in co-design, even though users may not necessarily think in terms of implications or risks, but rather in terms of conditions before the technology can be used. At the same time, this case study indicates that a co-design process that focuses on opportunities rather than balancing attention for both positive and negative implications may result in knowledge gaps related to implications and how they can be addressed. In the pursuit of responsible outcomes, co-design facilitators could consider broadening the scope of co-design processes, for instance, by moving back and forth between opportunities and associated implications of innovation, involving future users and social advocacy groups in such an inquiry, and ensuring that co-design participants are provided with inspiration and have basic knowledge and skills to contribute to anticipating implications. Explicit *anticipation* of implications in co-design and broader *inclusion* of stakeholders in doing so increase opportunities for innovators to start addressing implications of innovation before the technology design has been locked in.

## Appendix

Multimedia Appendix 1 The responsible innovation survey.

## Abbreviations

AAL: Active and Assisted Living
AI: artificial intelligence
DSS: decision support system
HAAL: Healthy Ageing Eco-system for People With Dementia
PM: project member
RI: responsible innovation
