# Supplementary material for: Making Co-Design More Responsible: Case Study on the Development of an AI-Based Decision Support System in Dementia Care
Source: JMIR Hum Factors. 2024 Jul 31;11:e55961. doi: 10.2196/55961 (PMC11325107; doi:10.2196/55961)
Supplement: Multimedia Appendix 1 [file humanfactors_v11i1e55961_app1.docx]

# RI-Survey

**Responsible Innovation survey – HAAL consortium

Exploring the potential and desired impact of the AI-based HAAL dashboard**

**1. Introduction**In HAAL, we are developing a dashboard in which the data from the different AAL (point) solutions is combined, analysed and coherently presented or even transformed into recommendations to support caregivers in their decision-making. In this survey, we ask HAAL project partners about your views and expectations about the positive and negative impacts of the HAAL dashboard on end-users. We use two scenarios about the HAAL dashboard to explore and define the role of artificial intelligence (AI) and its impact, as well as directions for further development. Also, we ask you about principles for responsible AI innovation and how we can promote these principles in the design and implementation of the HAAL dashboard. 

Through this survey, we aim to learn about your perspectives on innovation that is meaningful and responsible for end-users, i.e. people with dementia and their formal and informal caregivers. Please note that we ask you to answer the survey from your own perspective, so from your role in the HAAL consortium.

By completing this survey you give permission for the processing of the data by [blinded]. Preferably you do not include person-specific information such as your name in your answers, except for the last section of the survey, where you can optionally leave person-specific information. Your personal information can help us to track your views on responsible innovation in HAAL over time, and to compare the views of different HAAL project partners. The results will be used (anonymised where possible) as input for further research and development within the HAAL project. The results may also be used (anonymously) for publications. More information about the processing of data by [blinded] can be found in the [privacy statement](https://www.vilans.org/about-vilans/privacy-policy/) of [blinded]

This survey contains 10 sections. This is the end of section 1.

**2. Artificial intelligence**Importantly, the HAAL dashboard will be driven by artificial intelligence (AI). Artificial intelligence is the attempt to mimic parts of human intelligence in machines. A specific definition from the OECD states that AI technologies are *“machine-based systems that can, for a given set of human-defined objectives, make predictions, recommendations, or decisions influencing the real or virtual environments they interact with”*. Both pre-programmed (rule-based) algorithms and self-learning (data-based or machine learning) algorithms can be at the basis of AI technologies. 

The increasing advancements of AI-technologies such as the HAAL dashboard come with benefits such as better, faster and more efficient data analysis and decision-making, but also with challenges from a social and ethical perspective, e.g. related to privacy, transparency, human control and trust. Therefore, we are exploring **what decisions and actions can be taken in the design and implementation of the HAAL dashboard to achieve responsible innovation** (RI) that is socially desirable, ethically acceptable and sustainable.

Vr2_1: **Question: 
How do you feel about the increasing role of data and AI in healthcare and society (e.g. hopeful, suspicious, or otherwise)?
Please explain your answer.**

______________________________

**3. Types of AI-based analyses** 

Through an iterative user-centered design approach, the HAAL consortium is carefully determining what kind of overviews and AI-based analyses the dashboard should provide. Finally, the HAAL dashboard could provide descriptive, diagnostic, predictive and even prescriptive analyses. In the following, it is briefly explained what these types of analyses could entail in HAAL:
•    Descriptive analyses could mean that data from the various AAL products about the current situation in the client’s home is displayed and eventually translated into a coherent and comprehensible overview of insights. The data and insights can be about the client’s behaviour, health, well-being and safety, i.e. exercising, nutrition, medication intake and social contact.
•    Diagnostic analyses could mean that data gathered by several HAAL products is combined to create new meanings. For example, when data shows that the client’s sleep quality during the night is declining while activity during the day also declines, then the system may automatically link and display these two pieces of information together, as a way to imply that the day-night rhythm of the senior may be disturbed. 
•    Predictive analyses could mean that based on the data from HAAL products warnings or risk scores are given about possible future situations or emergencies such as a fall.
•    Prescriptive analyses could mean that recommendations are derived from the data that prompt caregivers to take a certain action, for instance to stimulate physical activity if a client is has been relatively inactive over the last days.

**4. Two scenarios about the dashboard**In this survey, we ask you to reflect on two scenarios about the HAAL dashboard in order to learn more about what is feasible, desirable and (ethically) acceptable. As shown in the figure below, the scenarios focus on different types of AI-based analyses. Scenario A primarily focuses on descriptive analyses with relatively low complexity and a low level of automation, as relatively much human input is required to translate these analyses into real-world decisions and actions. Scenario B provides various types of analyses (i.e. descriptive, diagnostic, predictive and even some prescriptive analyses) that involve more complexity and a higher level of automation. 

We are very interested in your views and opinions about both scenarios. After showing each scenario, we will ask you questions about it. 

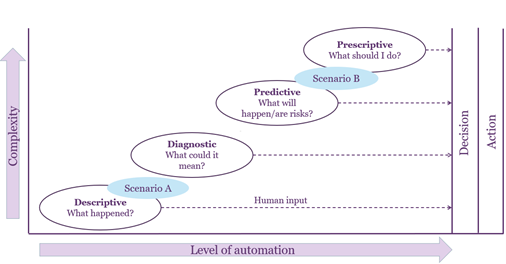

*Figure: Types of AI-based analyses*

**5. Scenario A: an integrated dashboard with a descriptive overview**The HAAL dashboard is a graphic user interface that provides professional caregivers a central entrance point to descriptive analyses about the behaviour, health, well-being and safety of their clients. The analyses are based on the data that is gathered through various AAL products that are deployed in the individual senior’s home setting. In the dashboard, caregivers can assess current situation and how this has changed over time.

**Examples**For instance, prior to a home visit to a client a caregiver looks into the dashboard. In a graph, he sees that for the last few days, the amount of physical activity of the client has been lower than usual. The caregiver interprets this as a health risk and assumes it will be good to stimulate physical activity. The caregiver arranges that the client is taken out for a walk the next day.

For another client the caregiver sees a notification in the dashboard that the client is not getting out of bed, while being awake for a few hours. This has been happening for multiple days in a row now. Also, the data shows that the client is lying in the same position during most of the time in bed. The caregiver wonders if something might be going on and decides to ask the client about it during the next visit, and to assess the risk for bed sores.

**Supportive system**Overall, the insights from the HAAL dashboard can support caregivers when making decisions about the care and support for a specific client, or at least provide starting points for further investigation. The dashboard is a low-complex system that can be easily understood and interpreted by caregivers. 

**Pre-programmed system**The HAAL dashboard does not simply show all the data that is gathered through the technologies. Only the information that is deemed to be most relevant at that moment is shown. In order to distil relevant insights from across the datasets, the dashboard uses algorithms that are defined by a team of human programmers and professionals caregivers. In addition, caregivers that use the platform have some options for manually tweaking the rules, in order to better tailor the dashboard to the context of the care organisation or a specific client. For instance, they could decide to let the dashboard show more detailed information about physical activity, or to prolong the period over which data is shown.

**HAAL bundle adjustments**In the dashboard, caregivers can also see what other technologies are available and could be connected to the platform. Through the dashboard, caregivers can manually initiate or stop the use of a particular technology. The purchase, installation, user training and other necessary steps are then automatically initiated and coordinated.

Vr5_1: **Questions:
1. What do you think about the added value of this dashboard for professional caregivers and clients?**

______________________________

I don’t know

Vr5_2: **2. What do you think could be the role of the informal caregiver in the use of the dashboard?**

______________________________

I don’t know

Vr5_3: **3. What are the main strengths of this scenario in your opinion?**

______________________________

I don’t know

Vr5_4: **4. What weaknesses do you (fore)see for caregivers in this scenario?**

______________________________

I don’t know

Vr5_5: **5. What weaknesses do you (fore)see for clients in this scenario?**

______________________________

I don’t know

Vr5_6: **6. What suggestions do you have about how to deal with these weaknesses?**

______________________________

I don’t know

**6. Scenario B: a forward-looking decision aid**The HAAL dashboard is a personal assistant that provides professional caregivers with real-time insights and predictions about the well-being, safety and health of clients. Also the dashboard gives recommendations for next steps in the care and support of these clients. The personal assistant provides these insights and recommendations both proactively and on demand of the caregiver, and can be interacted with through voice. 

**Examples**For instance, in the morning, the HAAL dashboard notifies to a caregiver that five of her clients might need closer attention based on erratic behaviour patterns in the past week. After looking briefly at graphs about these clients’ behavioural patterns, the caregiver quickly decides that three of the five clients need an urgent home visit. Through the HAAL dashboard, her working time on the screening process is shortened. Besides, more time is devoted to personalised care for clients with high risks.

During, and sometimes before home visits, the case manager looks into the HAAL dashboard to assess the situation of specific clients.  For one client, the platform displays that the client takes more time to get out of bed in the past 2 months. There is an orange flag next to the graph about the time for ‘rising-getting out’. The system comments that this indicates a high risk for a fall, and suggests a leg rehabilitation program to reduce the risk. During further talks with the client at home, the case manager decides to refer to a physiotherapist for a fall prevention program. 

**Supportive system**Overall, the HAAL dashboard relocates the workload for caregivers from intensive rehabilitation to prevention, for instance by helping to avert the risk of health complications that occur after a fall.

**Adaptive system**Over time more and more data are collected about the individual client, whereby the system's performances and insights verifiably become more and more reliable. Based on a combination of pre-programmed and self-learning algorithms, the dashboard is automatically interpreting all data, learning and reasoning about the client’s individual situation, and providing suggestions about the care situation and the best courses of action. Based on newly gathered data, the system is able to update its internal decision-making logic over time. In other words, the system is continuously improving the rules that are used to process raw data into relevant insights. At the same time, the rules used by the system become more difficult to explain over time, as it is not transparent how the system processes input to certain output.

**HAAL bundle adjustments**The AI-based dashboard proactively supports caregivers in adapting the eco-system of AAL technologies to the current needs of the older adult and his/her care network. Based on the data from the various HAAL products, the dashboard proactively provides suggestions to caregivers about care technologies that cannot or need not be used anymore, and other technologies and services that could be introduced to better support the situation at that stage. Through the dashboard, caregivers only need to approve or reject suggestions.

Vr6_1: **Questions:
1. What do you think about the added value of this dashboard for professional caregivers and clients?**

______________________________

I don’t know

Vr6_2: **2. What do you think could be the role of the informal caregiver in the use of the dashboard?**

______________________________

I don’t know

Vr6_3: **3. What are the main strengths of this scenario in your opinion?**

______________________________

I don’t know

Vr6_4: **4. What weaknesses do you (fore)see for caregivers in this scenario?**

______________________________

I don’t know

Vr6_5: **5. What weaknesses do you (fore)see for clients in this scenario?**

______________________________

I don’t know

Vr6_6: **6. What suggestions do you have about how to deal with these weaknesses?**

______________________________

I don’t know

**7. Comparison**

Vr7_1: **1. Which scenario is most desirable from a societal perspective, in your opinion?**

- Scenario A: an integrated dashboard with a descriptive overview
- Scenario B: a forward-looking decision aid

Vr7_1_Elab: **Please elaborate why.**

______________________________

Vr7_2: **2. Which scenario do you think is most ethically acceptable?**

- Scenario A: an integrated dashboard with a descriptive overview
- Scenario B: a forward-looking decision aid

Vr7_2_Elab: **Please elaborate why.**

______________________________

Vr7_3: **3. Which of the two scenarios do you expect is most feasible from the HAAL project perspective?**

- Scenario A: an integrated dashboard with a descriptive overview
- Scenario B: a forward-looking decision aid

Vr7_3_Elab: **Please elaborate why.**

______________________________

Vr7_4: **4. If the HAAL project would need to choose either scenario A or scenario B to focus on in further developments, which one would you then prefer?**

- Scenario A: an integrated dashboard with a descriptive overview
- Scenario B: a forward-looking decision aid

Vr7_4_Elab: **Please elaborate why.**

______________________________

**8. Principles for responsible AI innovation**Next, we will explore how a set of values and principles for responsible AI innovation can be promoted in the design and implementation of the HAAL dashboard. Values and principles can be seen as conceptions of what is good, proper, and desirable and as beliefs or moral compasses that could guide responsible innovation. Many organisations have already produced statements of the values or principles that should guide the development and deployment of AI in society. In HAAL, we strive to move the dialogue forward, from values and principles, to concrete recommendations about how to apply these principles in the project. In the following, we therefore present a set of principles for responsible AI innovation (adopted from Guidance on Ethics and governance of artificial intelligence for health from the World Health Organization) and we ask you to reflect on these principles in the light of the two scenarios. 

*When answering the questions, it could help to consider if certain elements from scenario A or B could either promote or undermine this principle. This may provide directions along which to think about measures that could be taken to ensure that this principle is promoted. At the same time, all suggestions are welcome, also apart from the scenarios.*

Vr8_1: **Principle 1: Autonomy**Adoption of AI can lead to situations in which decision-making could be or is in fact transferred to machines. The principle of autonomy requires that any extension of machine autonomy not undermines human autonomy. In the context of healthcare, this means that humans should remain in full control of healthcare systems and medical or care-related decisions. AI systems should be designed to assist humans in making informed decisions, whether they be care providers or clients.

**Question: 
What are your suggestions for the design and/or implementation of the HAAL dashboard to promote the autonomy of clients and their (formal and informal) caregivers?**

______________________________

I have no suggestions

Vr8_2: **Principle 2: Well-being & safety**AI technologies should not harm people. They should satisfy regulatory requirements for safety, accuracy and efficacy before deployment, and measures should be in place to ensure quality control and quality improvements.

**Question: 
What are your suggestions for the design and/or implementation of the HAAL dashboard to promote the well-being and safety of clients and their (formal and informal) caregivers?**

______________________________

I have no suggestions

Vr8_3: **Principle 3: Transparency & explainability**AI should be intelligible or understandable to developers, users and regulators. AI technologies should be explainable to the extent possible and according to the capacity of those to whom the explanation is directed. Many AI technologies are complex, and the complexity might frustrate both the explainer and the person receiving the explanation. There is a possible trade-off between full explainability of an algorithm (at the cost of accuracy) and improved accuracy (at the cost of explainability).

**Question: 
What are your suggestions for the design and/or implementation of the HAAL dashboard to promote the transparency and explainability of insights and outcomes shown by the HAAL-dashboard?**

______________________________

I have no suggestions

Vr8_4: **Principle 4: Responsibility & accountability**Although AI technologies perform specific tasks, it is the responsibility of human stakeholders to ensure that they can perform those tasks and that they are used under appropriate conditions. And when something does go wrong in application of an AI technology, there should be accountability.

**Question: 
What are your suggestions for the design and/or implementation of the HAAL dashboard to promote human responsibility and accountability?**

______________________________

I have no suggestions

Vr8_5: **Principle 5: Inclusiveness and equity**Inclusiveness requires that AI used in health care is designed to encourage the widest possible appropriate, equitable use and access, irrespective of age, gender, income, ability or other characteristics. AI technologies should be designed by and evaluated with the active participation of those who are required to use the system or will be affected by it, including providers and patients, and such participants should be sufficiently diverse. This means, for instance, that AI technologies should not be biased. Bias is a threat to inclusiveness and equity because it represents a departure, often arbitrary, from equal treatment.

**Question: 
What are your suggestions for the design and/or implementation of the HAAL dashboard to promote inclusiveness and equity?**

______________________________

I have no suggestions

Vr8_6: **Principle 6: Responsiveness**Responsiveness requires that designers, developers and users continuously, systematically and transparently examine an AI technology to determine whether it is responding adequately, appropriately and according to communicated expectations and requirements in the context in which it is used. For instance, the identification of a health need requires a response to that need. Or when an AI technology is ineffective or engenders dissatisfaction, there is a duty to be responsive by resolving the problem or even terminating the use of the technology. Also, AI technologies should be introduced only if they can be fully integrated and sustained in the health-care system.

**Question: 
What are your suggestions for the design and/or implementation of the HAAL dashboard to promote responsiveness of both the HAAL project and its outcomes (i.e. the AI-based HAAL dashboard)?**

______________________________

I have no suggestions

**9. Prioritizing the principles** 

Below we ask to rank the principles you saw in section 8 three times. Make sure you drag the answers up so the answer is green outlined.

Vr9_1: **1. Which of these principles is in your view most important to pay attention to in HAAL? 
Please rank them from most important to least important.**

| Principle 1: Autonomy |
| --- |
| Principle 2: Well-being & safety |
| Principle 3: Transparency & explainability |
| Principle 4: Responsibility & accountability |
| Principle 5: Inclusiveness and equity |
| Principle 6: Responsiveness |

Vr9_1_Expl: **Can you explain your prioritization?**

______________________________

I am not able to explain my prioritization

Vr9_2: **2. Which of the principles do you expect is hardest to achieve in HAAL?
Please rank them from hardest to easiest to achieve.**

| Principle 1: Autonomy |
| --- |
| Principle 2: Well-being & safety |
| Principle 3: Transparency & explainability |
| Principle 4: Responsibility & accountability |
| Principle 5: Inclusiveness and equity |
| Principle 6: Responsiveness |

Vr9_2_Expl: **Can you explain your prioritization?**

______________________________

I am not able to explain my prioritization

Vr9_3: **3. In relation to which principles do you see the largest risks if no explicit action is taken by the HAAL consortium to actively promote these principles? Please rank them from highest risk to lowest risk.**

| Principle 1: Autonomy |
| --- |
| Principle 2: Well-being & safety |
| Principle 3: Transparency & explainability |
| Principle 4: Responsibility & accountability |
| Principle 5: Inclusiveness and equity |
| Principle 6: Responsiveness |

Vr9_3_Expl: **Can you explain your prioritization?**

______________________________

I am not able to explain my prioritization

**10. Final questions**

Vr10_1: **1. What is your name?***(optional)*This is relevant for us to know, as we may ask you more questions later in the project, and this allows for a comparison over time. However, you can always decide to stay anonymous.

______________________________

Vr10_2: **2. For which partner organisation of the HAAL project do you work?** *(optional)*

______________________________

Vr10_3: **3. Can you briefly describe your function?***(obligatory question)*

______________________________

Vr10_4: **4. What is your age?***(optional)*

- ≤ 24 years
- 25 - 34 years
- 35 - 44 years
- 45 - 54 years
- 55 - 64 years
- 65 - 74 years
- 75 - 84 years
- ≥ 85 years

Vr10_5: **5. What HAAL work package(s) do you mostly work on?***(optional)
Multiple answers possible*

- WP1
- WP2
- WP3
- WP4
- WP5
- other, namely:: ______________________________

Thank you, your answers were saved perfectly.
